# Draft genome sequences of 12 triclosan tolerant bacteria isolated from returned activated sewage sludge

**DOI:** 10.1128/MRA.00336-23

**Published:** 2023-09-27

**Authors:** Sophie van Hamelsveld, Erin McGill, William Taylor, Louise Weaver

**Affiliations:** 1 Institute of Environmental Sciences Ltd., Christchurch, Canterbury, New Zealand; Department of Biological Sciences, Wellesley College, Wellesley, Massachusetts, USA

**Keywords:** triclosan, wastewater treatment, sewage sludge, antibiotic resistance, emerging organic contaminants

## Abstract

Herein we report the whole genome sequences of 12 highly triclosan tolerant bacteria isolated from returned activated sludge spiked with triclosan.

## ANNOUNCEMENT

The antibacterial additive triclosan has been detected in seawater and waste effluent (1), where it is toxic to microorganisms and eukaryotes (2–4). The use of bacteria for biodegradation of triclosan has been investigated (5–7). We selected 12 triclosan-tolerant strains isolated from returned activated sludge (RAS) for whole genome sequencing. Our aim was to provide taxonomic identification and to investigate the genetic mechanisms underpinning the strains’ triclosan tolerance.

The strains were isolated from RAS collected from a municipal wastewater treatment plant in Aotearoa, New Zealand. Samples were mixed and pelleted by centrifugation. Pellets were spiked with triclosan to give a final concentration of 5 mg/L triclosan. Replicate samples were maintained in darkness at 20°C with aeration. After 40 d, dilutions were plated on M9 agar containing triclosan (5 mg/L) and incubated at 20°C for 7 d. After 7 d, well-isolated colonies were picked, subcultured, and grown on fresh minimal medium + triclosan (5 mg/L). After 7 d, these were again subcultured in minimal medium +triclosan agar and minimal medium +triclosan broth. Twelve isolates were able to grow in both media, and all of these were progressed for sequencing.

DNA was extracted from single colonies cultured on tryptic soy agar using the Qiagen DNeasy PowerSoil Pro Kit according to the manufacturer’s instructions. Sequencing libraries were prepared using the Nextera XT v2 Library Prep Kit (Illumina) according to the manufacturer’s instructions. On average, 1,707,389 Illumina 2 × 150 bp reads (NextSeq 550, Environmental Science Research Ltd., Porirua, New Zealand) were produced at an average depth of 71.3 × per isolate. Quality control was performed with fastqc v0.11.7 (https://www.bioinformatics.babraham.ac.uk/projects/fastqc/) and trimming and adapter removal with bbduk v38.90 (sourceforge.net/projects/bbmap/). The assemblies were produced with Skesa v.2.3.0 (8). Species information was generated using Kraken v2.1.2 (9) and fastANI v1.33 (10). Triclosan-degradation and antimicrobial resistance genes were annotated using ABRicate v.1.0.1 (https://github.com/tseemann/ABRicate), with parameters —minid 50 —mincov 50. Public-facing genomes were annotated by PGAP v5.3 (11). Unless stated, the software was run with default parameters. Sequencing metrics and strain information are presented in Table 1.

**TABLE 1:** Genome statistics and genotyping information^*a*^

Strain	Species	ANI	Accession	No. of reads (million)	Genome length (bp)	N50 (bp)	Coverage depth (×)	GC content	Contigs (n)	BioSample accession no.	SRA accession no.	Putative triclosan tolerance genes
GW210006_S54	*Cupriavidus basilensis*	88.23	GCA_008801925.2	1.2	9,329,027	21,712	36	64.71%	739	SAMN32359043	SRR22859034	*axyY, mexBDK-oprM, muxBC-opmB*
GW210007_S55	*Pseudomonas putida*	90.29	GCF_000412675.1	2	5,934,976	45,348	98	61.70%	232	SAMN32359044	SRR22859033	*mexJK-opmH, mexEF-oprN, muxABC-opmB, triABC, rsmA*
GW210008_S56	*Achromobacter denitrificans*	99.16	GCF_013267375.1	2.2	6,544,063	22,859	98	67.57%	449	SAMN32359045	SRR22859032	*axyXY-oprZ, mexAB-oprM, parRS, opcM*
GW210009_S57	*Pseudomonas denitrificans*	94.22	GCA_008807415.1	1	6,527,965	32,189	45	64.46%	368	SAMN32359046	SRR22859031	*mexAB-oprM, mexBDK,oprM, muxABC-opmB, opcM*
GW210010_S58	*Cupriavidus basilensis*	87.00	GCA_008801925.2	1.3	9,615,976	23,524	39	65.48%	718	SAMN32359047	SRR22859030	*mexAB-oprM, mexEF-oprN, mexJK-opmH, mexVW-oprM, muxABC-opmB, pmpM, emrE, soxR, basSR, rosB, rsmA*
GW210011_S59	*Pseudomonas putida*	98.32	GCF_000412675.1	1.1	6,596,672	28,282	50	62.00%	462	SAMN32359048	SRR22859029	*mexAB-oprM, mexEF-oprN, mexJK-opmH, mexVW-oprM, mexMN-oprM, muxABC-opmB, cpxR, emrE, soxR, triABC, rsmA*
GW210012_S60	*Pseudomonas putida*	98.32	GCF_000412675.1	1.2	6,600,046	26,290	53	62.00%	447	SAMN32359049	SRR22859028	*mexAB-oprM, mexEF-oprN, mexJK-opmH, mexVW-oprM, mexMN-oprM, muxABC-opmB, cpxR, emrE, soxR, triABC, rsmA*
GW210013_S61	*Achromobacter denitrificans*	99.12	GCA_013267375.1	1.1	6,599,651	16,954	49	67.53%	631	SAMN32359050	SRR22993526	*axyXY-oprZ, mexAB-oprM, mexJK, muxABC-opmB, opcM*
GW210014_S62	*Achromobacter denitrificans*	99.09	GCA_013267375.1	2.1	6,456,411	22,619	91	67.52%	463	SAMN32359051	SRR22993525	*axyXY-oprZ, mexAB-oprM, mexJK, muxABC-opmB, opcM*
GW210015_S63	*Pseudomonas citronellolis*	98.03	GCF_001586155.1	2.4	7,058,728	22,356	95	67.15%	515	SAMN32359052	SRR22993524	*mexAB-oprM, mexEF-oprN, mexJK-opmH, mexVW-oprM, muxABC-opmB, pmpM, emrE, soxR, basS, rosB, rsmA*
GW210017_S64	*Achromobacter anxifer*	98.89	GCA_903652925.1	2.4	7,111,444	43,579	100	66.21%	277	SAMN32359053	SRR22993523	*axyXY-oprZ, mexAB-oprM, mexCD-oprJ, mexI, mexK, muxABC-opmB*
GW210018_S65	*Pseudomonas citronellolis*	91.39	GCF_001586155.1	2.4	6,769,216	31,062	102	67.48%	397	SAMN32359054	SRR22993522	*mexAB-oprM, mexEF-oprN, mexJK-opmH, mexVW-oprM, muxABC-opmB, pmpM, emrE, soxR, basS, mdtN, rosB, rsmA*

^*a*^ Sequence read archive data and assemblies have been deposited under BioProject PRJNA914847. ANI: average nucleotide identity, SRA: sequence read archive*.*

These isolates represent three genera: *Pseudomonas* (6)*, Cupriavidus* (2), and *Achromobacter* (4). Operons encoding multidrug efflux pumps such as *mex, mux,* and *tri* were frequently annotated, possibly accounting for the strains’ high triclosan tolerance (12–14). Antimicrobial resistance genes such as *sul*2 and *aph*(6)-lc were also present in one isolate each (data not shown). It has previously been demonstrated that some bacteria can utilize triclosan as a sole carbon source (15) but the mechanisms used remain unclear.

Our results agree with previous studies suggesting that the genera *Pseudomonas*, *Achromobacter,* and *Cupriavidus* are degraders of triclosan (12, 14–17). Further research to assess the ability of these strains to degrade triclosan is planned.

## Data Availability

The data have been deposited in GenBank under BioProject PRJNA914847, and the accession numbers are available in Table 1.
